# Prevalence of G6PD deficiency and molecular characterization of G202A, A376G and C563T polymorphisms in newborns in Southeastern Brazil

**DOI:** 10.31744/einstein_journal/2019AO4436

**Published:** 2019-01-17

**Authors:** Lucas Luís Meigre Dias Pereira, Cristina Augusta Bravin, Terezinha Sarquis Cintra, Wélida Santos Portela Cassa, Thainá Altoé Santos, Armando Fonseca, Rodrigo Pratte-Santos

**Affiliations:** 1 Faculdade PIO XII , Cariacica , ES , Brazil .; 2 Associação de Pais e Amigos dos Excepcionais , Vitória , ES , Brazil .; 3 Laboratório de Genética do Espírito Santo , Vitória , ES , Brazil .; 4 Diagnósticos Laboratoriais Especializados Ltda , São Paulo , SP , Brazil .; 5 Universidade Federal do Espírito Santo , Vitória , ES , Brazil .

**Keywords:** Glucosephosphate dehydrogenase deficiency, Polymorphism, genetic, Infant, newborn, Deficiência de glucosefosfato desidrogenase, Polimorfismo genético, Recém-nascido

## Abstract

**Objective:**

To evaluate the prevalence of G6PD deficiency and characterize G202A, A376G and C563T polymorphisms in neonates using molecular assays.

**Methods:**

A total of one thousand samples were tested through quantitative analysis of enzyme activity, detecting 25 G6PD-deficient individuals. Patients identified as deficient were submitted to molecular analysis quantitative real-time polymerase chain reaction – (qPCR) to investigate the presence of variants associated with the deficiency.

**Results:**

The total prevalence of G6PD deficient was 2.5%. Of the 25 samples identified as deficient, 21 were submitted to qPCR assay to analyze the presence of G202A, A376G and C563T variants. All samples showed the G202A/A376G genotype, characterizing G6PD A- phenotype.

**Conclusion:**

The prevalence of G6PD deficiency in the present study was similar to that observed in other study populations in Brazil. Molecular analysis identified in all patients the presence of the genetic polymorphism G202A/A376G, more common in the Brazilian population with G6PD deficiency, which is directly estimated by enzyme activity level.

## RESUMO

**Objetivo:**

Avaliar a prevalência da deficiência de G6PD e caracterizar, por ensaios moleculares, os polimorfismos G202A, A376G e C563T em recém-nascidos.

**Métodos:**

Foram testadas mil amostras por meio da análise quantitativa da atividade enzimática, detectando 25 portadores de deficiência de G6PD. Os pacientes identificados como deficientes foram submetidos à análise molecular reação em cadeia da polimerase em tempo real (qPCR) para pesquisa da presença das variantes associadas à deficiência.

**Resultados:**

A prevalência total de deficientes de G6PD foi de 2,5%. Das 25 amostras identificadas como deficientes, 21 foram submetidas ao qPCR, para análise da presença das variantes G202A, A376G e C563T. Todas as amostras apresentaram o genótipo G202A/A376G, caracterizando fenótipo G6PD A-.

**Conclusão:**

A prevalência da deficiência da G6PD no presente estudo foi semelhante à verificada em outras populações de estudo no Brasil. A análise molecular identificou em todos os pacientes a presença do polimorfismo genético G202A/A376G, mais comum na população brasileira portadora da deficiência de G6PD, que é diretamente estimada pelo nível de atividade enzimática.

## INTRODUCTION

Glucose-6-phosphate dehydrogenase (G6PD) deficiency is a genetic disorder and affects more than 400 million people in the world. ^(^ ^1^ ^-^ ^3^ ^)^ G6PD-deficient patients are mostly asymptomatic and may have crises when exposed to situations that cause oxidative stress, such as ingestion of fava beans, infections, and use of medications. Such crises are identified by onset of some symptoms, such as malaise, abdominal or low back pain, weakness, jaundice, and dark urine, ^(^ ^2^ ^)^ which characterize acute oxidative hemolysis, neonatal jaundice or chronic nonspherocytic hemolytic anemia. ^(^ ^2^ ^)^ Factors influencing the severity and frequency of episodes of expression of this deficiency are strictly associated to the relation of genetic and environmental factors with the molecular properties of enzymes of the patients. ^(^ ^4^ ^)^

Glucose-6-phosphate dehydrogenase comprises 514 amino acids, with an approximate molecular weight of 59 kDa, present in all cells of the body. This enzyme participates in the first step of the hexose monophosphate pathway, oxidizing glucose-6-phosphate to 6-phosphogluconolactone, reducing NADP to NADPH, which is an important mediator of oxidative stress in the cell. ^(^ ^2^ ^,^ ^5^ ^,^ ^6^ ^)^ This pathway is the only one to obtain NADPH in erythrocytes in order to protect them from oxidative stress. ^(^ ^7^ ^)^ Another function attributed to the enzyme is to obtain energy, since the pentose-phosphate pathway catabolizes about 10% of glucose in the cell. The remaining 90% are catabolized via the Embden-Meyerhof pathway. ^(^ ^8^ ^)^

There are approximately 186 clinically relevant mutations reported for the G6PD gene, which encode deficient variants of the physiologically normal enzyme. Class I variants, such as G6PD volendam (C514T), characterize severe deficiency and are associated with chronic nonspherocytic hemolytic anemia. Class II variants, such as G6PD Mediterranean (C563T), have enzyme activity values lower than 10%, leading to more frequent symptoms and crises. In class III variants, including G6PD A (A376G), A- (G202A/A376G), G6PD Asahi (G202A), the enzyme activity ranges from 10 to 60%, and patients are usually asymptomatic, but the clinical relevance is the expression of medication-induced hemolytic crises. ^(^ ^6^ ^,^ ^9^ ^-^ ^11^ ^)^ Class IV variants, such as G6PD São Borja (G337A), have 60 to 150% of normal enzyme activity, with no clinical manifestations. Class V variants have increased enzyme activity (>150%) and were described only once (G6PD Hektoen), in 1969. ^(^ ^10^ ^-^ ^12^ ^)^ The most common phenotypes are G6PD A (genotype A376G), which has normal enzyme activity; G6PD A- (genotype G202A), which presents from 10 to 60% of the normal enzyme activity; and B- or G6PD Mediterranean (genotype C563T), with 7% of normal activity. ^(^ ^4^ ^,^ ^13^ ^)^ The G6PD-encoding gene is located in the telomere of the long arm of X chromosome (Xq28). This gene spans approximately 18.5 kb and contains 13 exons. ^(^ ^2^ ^)^

## OBJECTIVE

To evaluate the prevalence of G6PD deficiency in neonates considering enzyme activity, and characterize G202A, A376G, and C563T polymorphisms in neonates identified as enzyme-deficient.

## METHODS

**Figure 1:**
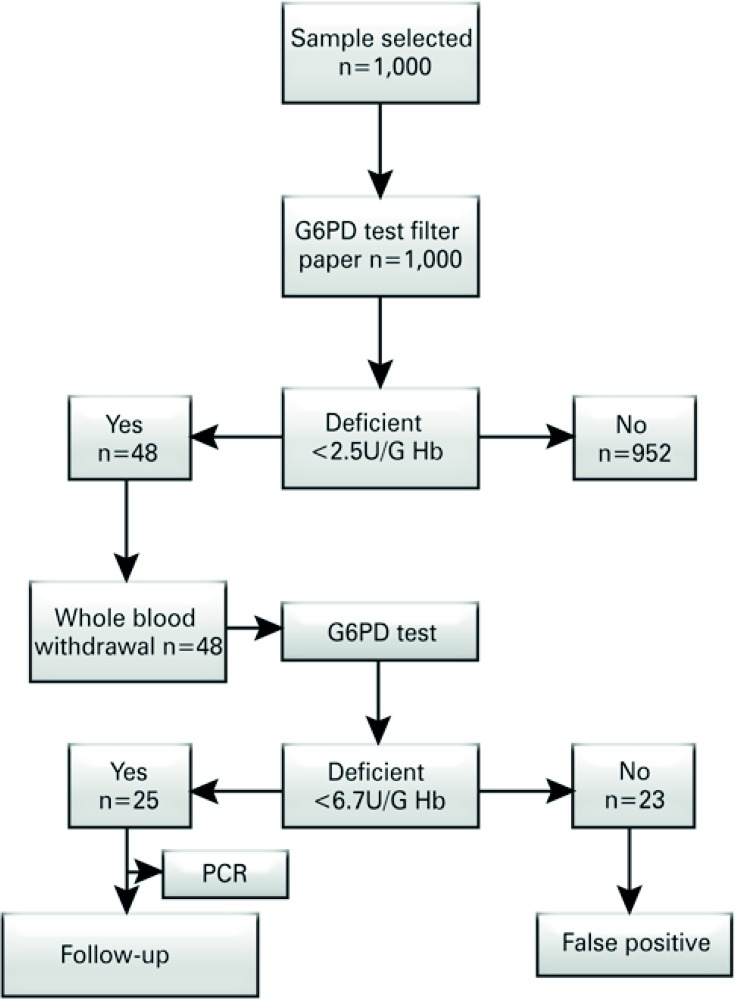
Study design for screening and identification of glucose-6-phosphate dehydrogenase (G6PD) in newborns

The study was conducted in the neonatal screening service of the *Associação de Pais e Amigos dos Excepcionais* (APAE) [Association of Parents and Friends of Special Needs Individuals] of the city of Vitória (State of Espírito Santo, Brazil). It was based on the study on prevalence of G6PD deficiency in the metropolitan region of Vitória (ES), in the State of Espírito Santo, in Southeastern Brazil. The study design is depicted in the flowchart (  Figure 1  ).

Hb: hemoglobin; PCR: polymerase chain reaction

A total of one thousand blood samples were collected in order to quantify the enzyme activity for G6PD. After the primary analysis, the patients identified as possibly deficient were submitted to a confirmatory test using whole blood. After the confirmatory test, molecular analysis was conducted on the samples confirmed as deficient. The samples were collected on high absorption filter paper for dry blood Whatman ^®^ 903, according to the Ordinance GM/MS 822 of June 6, 2001, and in a blood collection tube containing ethylenediaminetetraacetic acid (EDTA). ^(^ ^14^ ^)^

For this study, neonates from the metropolitan region of Vitória, State of Espírito Santo, born from May 24, 2017 to August 24, 2017, were accrued. The samples were selected based on the time between sample collection and arrival at the APAE, established as a maximum of 5 days; samples of newborns with up to 30 days of life and that corresponded to the baseline time between collection and arrival in the laboratory were accepted.

The enzymatic colorimetric method using commercial kit (INTERCIENTÍFICA ^®^ NeoLISA G6PD, São José dos Campos, SP, Brazil), was used to quantify the G6PD enzyme activity; the test was conducted according to the instructions of the manufacturer. Samples collected on filter paper were punched in single 1/8’’ or 3.2mm spots using an automatic puncher and palced in a 96-well microplate to conduct the analysis by absorbance using the DIAS ^®^ microplate reader, the interpretation of results being done using the TMS ^®^ software. The G6PD calculation software provided by INTERCIENTÍFICA ^®^ was used to calculate the enzyme activity based on the three readings. For the quantification of G6PD enzyme activity in the whole blood samples, the same commercial kit (INTERCIENTÍFICA ^®^ NeoLISA G6PD) was used, according to the manufacturer instructions. The cutoff value for normal results adopted in the filter paper test was >2.5U/G Hb. For the confirmatory test in whole blood, the cutoff value for normal activity was >6.7U/G Hb; intermediate values were between 2.2 and 6.6U/G Hb; and <2.1U/G Hb were considered deficient.

The A376G, G202A, and C563T mutations were genotyped by TaqMan™ (Life Technologies) assays using ABI 7500 real-time polymerase chain reaction (qPCR) (Applied Biosystems, California, USA). Fluorescence curves were analyzed with the 7500 FAST Sequence Detection Software version 2.1 (Applied Biosystems, California, USA) for allelic discrimination.

### Data analysis

The patients´ demographic data and G6PD enzyme activity were evaluated using Microsoft Excel spreadsheet, version 2016. The statistical comparison of G6PD enzyme deficiency with the demographic data was carried out by the χ ^2^ test with 5% significance level, using the statistical software Stata 11.0 (Stata Corp., College Station, USA).

### Ethical considerations

The study was approved by the Internal Review Board of the *Hospital Meridional* , under opinion number 2.362.064/2017, CAAE: 79236317.7.0000.5070.

## RESULTS

Of the 1,000 newborns screened at the APAE neonatal screening service in the city of Vitória, (ES) 48 (4.8%) were identified as possible carriers of the disease and referred to confirmatory tests by the colorimetric enzymatic method in whole blood samples. The prevalence of G6PD deficiency confirmed in this study was of 25 patients (2.5%).

Table 1  depicts the clinical and social characteristics of the study population. There were 508 male subjects (50.8%). All (n=25) patients with G6PD deficiency were male. The declared skin color of neonates enrolled in this study was distributed into three categories plus one of undeclared: 153 (15.3%) subjects were declared white by their parents or guardians; 454 (45.4%) brown; 92 (9.2%) black; and 301 (30.1%) had no declared color. The color declared for patients with G6PD deficiency did not present statistical difference (p=0.8938).

**Table 1:** Clinical and social characteristics of the study population

Characteristics	Normal G6PD (n=975)	Deficient G6PD (n=25)	Total (n=1,000)	p value
Sex	975 (100)	25 (100)	1,000 (100)	<0.0001
Female	492 (50.5)	0 (0)	492 (49.2)	
Male	483 (49.5)	25 (100)	508 (50.8)	
Age, months	Newborn	Newborn	-	-
Self-declared skin color				0.8938
White	149 (15.3)	4 (16)	153 (15.3)	
Brown	441 (45.2)	13 (52)	454 (45.4)	
Black	90 (9.2)	2 (8)	92 (9.2)	
Not declared	295 (30.3)	6 (24)	301 (30.1)	
Body mass, g	3094.3±846.8	3285.1±492.7	-	-
Delivery				0.9193
Term	953 (97.7)	24 (96)	977 (97.7)	
Preterm	22 (2.3)	1 (4)	23 (2.3)	
Maternal use of corticosteroids*				0.0062
Yes	7 (0.7)	2 (8)	9 (0.9)	
No	968 (99.3)	23 (92)	991 (99.1)	

Results expressed as n (%). * within 15 days before delivery. G6PD: glucose-6-phosphate dehydrogenase.

The enzyme activity values for the deficient patients ranged from 1.56 to 5.29U/G Hb with a mean value of 3.01U/G Hb. Molecular assays were carried out in 21 of 25 patients by real-time PCR in order to characterize the genetic polymorphisms. G202A and A376G variants were hemizygous in all 21 (100%) patients. The percentage of enzyme activity found in the 21 patients was, on average, 49.25%, based on cutoff values for normal enzyme activity.

Statistical significance (p=0.0062) was found when comparing the group of enzyme-deficient patients and maternal corticosteroid administration in the last 15 days before delivery and those with deficiency with no maternal hormone administration.

## DISCUSSION

The prevalence value described as 2.5% is within the Brazilian standards for studies on the prevalence of G6PD deficiency. Studies with neonates in the states of Mato Grosso, Rio Grande do Sul, and Paraíba, with study populations ranging from 147 to 3,573 subjects tested, found a mean prevalence of 4.75%. ^(^ ^15^ ^-^ ^17^ ^)^ Other studies evaluating population prevalence for G6PD deficiency in Brazil among adults and children obtained a mean prevalence of 3.59%, varying between 1.70 and 4.96% of G6PD-deficient subjects. ^(^ ^18^ ^-^ ^26^ ^)^

All patients identified as having the disease in our study were male, corroborating the assertion that this enzymopathy is more common in males, considering that they have only one G6PD gene, located on the single X chromosome, hence hemizygous for this gene. ^(^ ^2^ ^)^ These subjects, therefore, may be deficient or not for G6PD. Females, however, have two genes for G6PD, located one on each X chromosome; they may be deficient, characterizing homozygosis, or intermediate, when the mutation is only present in one of the two genes, denoting heterozygosis. ^(^ ^2^ ^)^ Nonetheless, differently from common enzyme deficiencies, heterozygous subjects may develop characteristic symptoms for G6PD deficiency. ^(^ ^2^ ^)^

Molecular analysis was carried out in only 21 of the 25 deficient patients, since the remaining four patients were seen in the laboratory for the confirmatory test after the molecular assays had been started. Of the 21 tested, 100% presented hemizygous A376G and G202A polymorphisms, characterizing the G6PD A- phenotype, described as G6PD African. ^(^ ^7^ ^)^ This genotype is the result of several overlapping points, presenting the A376G mutations, which are characteristic of the G6PD A+ phenotype, and G202A. ^(^ ^7^ ^)^ The G202A mutation occurring homogeneously is not sufficient to cause enzyme deficiency, and requires association with the A376G mutation. ^(^ ^27^ ^)^ In contrast, a case study in Japan identified a 3-year-old male patient who presented symptoms compatible with G6PD deficiency. The enzyme activity showed values compatible with the G6PD deficiency and was followed by molecular study through PCR-SSCP, which identified the presence of G202A mutation. However, an associated mutation was not identified, and the enzyme deficiency in this patient was considered as caused by G202A mutation, in a homogenous manner. ^(^ ^28^ ^)^ Nevertheless, the technique used to detect the mutation in this study (PCR-SSCP) is efficient, but its sensitivity may be altered by some key factors, such as the size of the DNA fragment used, which could lead to unreliable results. ^(^ ^29^ ^)^

The enzyme activity for G6PD in the subjects of the present study was, on average, 48.71% of normal values, being within the values proposed for the G6PD A variant, ranging from 10 to 60% of the normal enzyme activity for this variant. ^(^ ^4^ ^)^ Patients are defined as class III variant, with 10 to 60% of normal activity. ^(^ ^6^ ^)^ The G6PD A- phenotype found in the study is commonly reported in studies conducted in Brazil. A large study carried out in 1993 aimed to determine the molecular characterization of the G6PD-deficient subjects throughout the Brazilian territory. It used a sample of 7,794 patients, and found a significant prevalence of G6PD A- phenotype among subjects declared as white or black; however, this mutation pattern was not observed in the Brazilian indigenous people tested. ^(^ ^30^ ^)^ A study performed in the state of Mato Grosso found the African G6PD phenotype in 58 of 63 neonates tested. ^(^ ^11^ ^)^ The analysis of results of these and other studies ^(^ ^8^ ^,^ ^14^ ^,^ ^30^ ^)^ corroborates the hypothesis that the mutational patterns or polymorphisms described in the present study are within the pattern found for the Brazilian territory.

The high prevalence of this phenotype in the Brazilian territory is related to the processes of colonization and the use of peoples of African origin as labor force in the colonial period, leading to broad miscegenation and, consequently, dissemination of their genetic characteristics.

However, this study showed difficulty in communicating to patients to return to the outpatient clinic and confirm the enzyme deficiency by means of diagnostic tests. This problem may be attributed to the fact that G6PD deficiency, despite being a relatively common enzymopathy, is little known by lay people, mainly because the patients with this deficiency are mostly asymptomatic. The percentage of false-positive results in this study after the confirmatory test was 47.91%. This may be related to preservation factors that interfere in the quality of the sample in filter paper, in the collecting sites, such as site of puncture, temperature, and form of deposition of the collection cards, altering the enzyme activity identified in the initial test. The confirmatory test is necessary to describe the presence or absence of the deficiency.

Patients who had a positive confirmatory test for G6PD deficiency were referred to the APAE geneticist to initiate treatment, and monitor the progression of symptoms.

During visits to the physician, it was verified whether corticosteroids had been administered to the mother during pregnancy. When administered to pregnant women who are at risk for preterm birth, corticosteroids are not likely to cross the placental barrier and be in direct contact with the fetus. ^(^ ^31^ ^)^ In the present study, the groups of deficient patients were compared as to maternal use of steroids, showing statistical significance. Nevertheless, the drug was administered within 15 days before delivery, to reduce the risk of premature birth, and could not be directly related to the development of G6PD deficiency. All enzyme-deficient patients from both groups (with and without use of steroids) were genetically tested, and presented the same genetic pattern characteristic for G6PD deficiency.

The side effects of prolonged administration and direct contact of the fetus with corticosteroids are related to liver abnormalities, growth deficits, and long-term abnormalities during growth. ^(^ ^31^ ^)^

Studies such as this are required to include G6PD deficiency in the neonatal screening program of the State of Espírito Santo, where the study was conducted. Failure to diagnose deficiency in the early stages of life can lead to the development of severe symptoms when exposed to risk factors, such as hemolytic crisis after taking some medications.

This study is extremely important for the public health system of the State of Espírito Santo, considering that it is the first investigation to characterize a G6PD-deficient population, opening doors to include this disease in the neonatal screening program, and make the diagnosis available in the public system. The test is currently available only in private clinics of the state and the price is not affordable for the population at large.

## CONCLUSION

This study found the prevalence in the population studied within the Brazilian epidemiologic standards for this disease. The molecular analysis of the deficient patients was performed to characterize the polymorphisms causing the deficiency, and used the panel of the three most common mutations, A376G, G202A, C563T. The result of the molecular characterization indicated that all deficient patients presented the G6PD A- phenotype, characterizing G202A A376G genotype, common for the Brazilian population.

Maternal use of steroids among deficient subjects was statistically significant, however it was not possible to relate this variable to the development of the genetically acquired G6PD deficiency.
